# IL-37 is associated with osteoarthritis disease activity and suppresses proinflammatory cytokines production in synovial cells

**DOI:** 10.1038/s41598-017-11397-5

**Published:** 2017-09-14

**Authors:** Liping Ding, Xiaoping Hong, Baodong Sun, Qin Huang, Xiaoqi Wang, Xiaokai Liu, Lingyun Li, Zhong Huang, Dongzhou Liu

**Affiliations:** 1 Department of Rheumatology and Immunology, Shenzhen People’s Hospital, the Second Clinical Medical College of Jinan University, Shenzhen, China; 2 Department of Immunology and Microbiology, Biological Therapy Institute, Shenzhen University School of Medicine, Shenzhen, China

## Abstract

The objective of this study is to investigate the correlation between IL-37 level and osteoarthritis activity and to determine the anti-inflammatory effects of IL-37 in peripheral blood mononuclear cells (PBMCs) and synovial cells (SCs) from osteoarthritis (OA) patients, which including 32 patients with erosive inflammatory OA (EIOA) and 40 patients with primary generalized OA (PGOA), 40 age and sex matched healthy volunteers were recruited as healthy controls (HCs). The protein and relative mRNA levels of IL-37 were significant increased in the blood of EIOA patients compared with those of PGOA patients and HCs. Serum IL-37 levels of OA patients were positively correlated with VAS score, as well as with CRP, ESR in blood. Positive correlations were also observed among IL-37 with IL-1β, TNF-α and IL-6 in synovial cells. Furthermore, the expression of IL-1β, TNF-α and IL-6 in PBMCs and SCs from EIOA patients was suppressed by IL-37 *in vitro*. In conclusion, our results indicated that IL-37 increased in EIOA patients and was positively correlated with disease activity, the pro-inflammatory cytokines such as IL-1β, TNF-α and IL-6 in PBMCs and synovial cells from EIOA patients were restrained by recombinant IL-37. Thus, IL-37 may serve as a novel therapeutic target for the treatment of OA inflammation.

## Introduction

Osteoarthritis (OA), a most common degenerative joint disease, is characterized by cartilage breakdown, the formation of osteophytes at joint margins and generalized aches and pains^1–3^. It is traditionally considered that aging, obesity, wear and tear, genetic factors and other systemic diseases might be related to the pathogenesis of OA^3–5^. Predisposing conditions can often be used to distinguish secondary osteoarthritis from primary osteoarthritis, but this classification criteria is by no means firm^6^. Osteoarthritis can be classified as primary generalized osteoarthritis (PGOA) and erosive inflammatory osteoarthritis (EIOA) according to various clinical symptom and inflammation^7^.

In recent years, many investigations have focused on studying how cytokines are related to inflammatory processes, especially synovitis in OA, which is associated with pain sensitization and progression of the OA disease^8, 9^. There is compelling evidence that various inflammatory cytokines participating in the pathogenesis of OA, and IL-1β, TNF-α and IL-6 are the most important pro-inflammatory mediators in the development of OA disease^10, 11^. Exist simultaneously, regulatory cytokines, which are related to the relief of synovitis and protect joint tissue from excessive inflammation in OA, have also gained considerable attention^12^. These anti- inflammatory cytokines, such as interleukin 4 (IL-4), IL-10 and TGF-β, protected joint tissues by suppressing aggressive inflammatory response in OA^13–15^.

Interleukin-37, a new member of the anti-inflammatory cytokines, widely suppresses innate immunity as well as acquired immune responses^16^. *In vivo*, IL-37 has been shown to suppress the production of cytokines involved in the inflammatory response in several autoimmune and inflammatory diseases, such as inflammation of ischemia-reperfusion injury, psoriasis, DSS colitis and obesity-induced inflammation^17–20^. *In vitro*, IL-37 reduced the expression of IL-1β, IL-8, IL-1α and TNF-α in THP-1 and A549 cells^21^. Recently, we have shown that IL-37 inhibits disease-related inflammatory cytokines in PBMCs of patients with systemic lupus erythematosus (SLE), Graves’ Disease(GD), ankylosing spondylitis (AS) and rheumatoid arthritis(RA)^22–25^. Although accumulated data suggest that IL-37 may play an important role in controlling inflammation of the above diseases, the relationship between the expression and function of IL-37 and OA is not clear. In this study, we examined the expression of IL-37 mRNA and protein in peripheral blood between PGOA and EIOA patients and investigated the correlation of IL-37 levels with clinical data in serum and pro-inflammatory mediators in synovium of EIOA. In addition, we analyzed the suppressive function of IL-37 on the pro-inflammatory cytokines (TNF-α, IL-1β, and IL-6) in PBMCs and SCs of EIOA patients.

## Results

### Increased IL-37 mRNA and protein levels in patients with erosive inflammatory OA, but not in primary generalized OA

**Figure 1:**
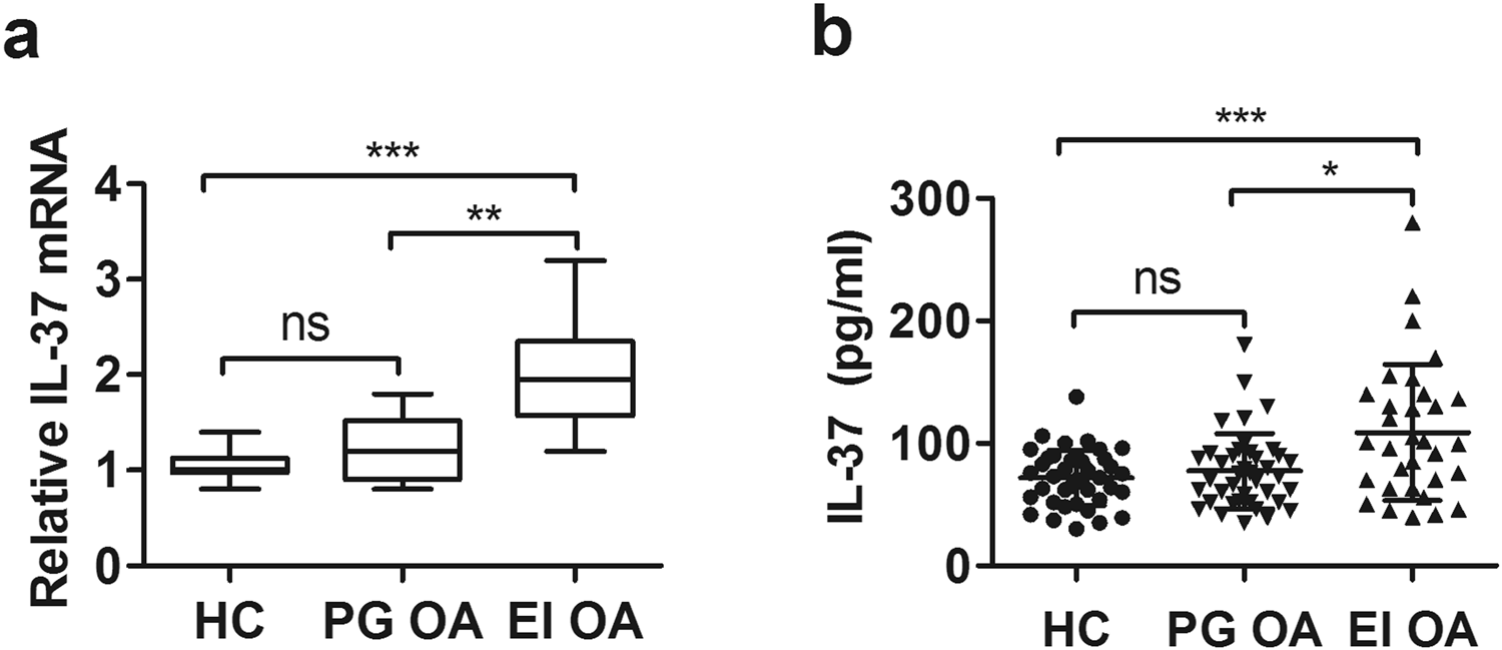
Comparison of IL-37 mRNAs and protein levels between OA and HC. Expression of IL-37 mRNAs in PBMCs from primary generalized OA (n = 40), erosive inflammatory OA (n = 32) OA patients and HCs (n = 40) was detected by RT-PCR **(a)**. Serum IL-37 protein levels were determined by ELISA **(b)**. Each symbol represents an individual OA patient and HC. The results are presented as mean ± SD.

32 patients with erosive inflammatory OA, 40 primary generalized OA patients and 40 age- and sex-matched HCs were enrolled. Compared with HCs, IL-37 mRNA levels were elevated notably in patients with erosive inflammatory OA (Fig. 1a). Serum protein levels of IL-37 were significantly higher in erosive inflammatory OA patients than in HCs (Fig. 1b). We also observed significantly increased IL-37 mRNA and serum levels in patients with erosive inflammatory OA compared to patients with primary generalized OA. No significant differences were observed in IL-37 mRNA and protein levels between primary generalized OA patients and healthy controls. Thus, these results suggested that IL-37 may be involved in the pathogenesis of erosive inflammatory OA.

### Correlation of IL-37 levels with clinical values

**Figure 2:**
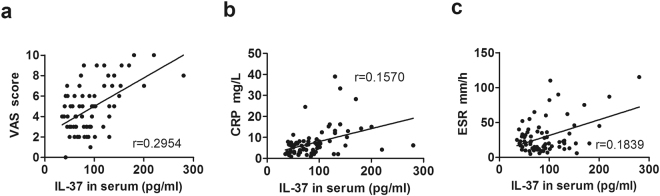
Correlations of serum IL-37 levels with OA clinical values. Each symbol represents an individual OA patient. Serum IL-37 levels in OA were positively correlated with VAS **(a)**, CRP **(b)** and ESR **(c)**. The correlations were evaluated with Spearman’s non-parametric test.

To determine the relationship between serum IL-37 levels and OA clinical indexes of inflammation, we detected the positive correlations of IL-37 with clinical values including VAS, CRP and ESR. As shown in Fig. 2, serum IL-37 levels were positively correlated with VAS (Fig. 2a, r = 0.2954), CRP (Fig. 2b, r = 0.1570), ESR (Fig. 2c, r = 0.1839).

### IL-37 inhibits the increased expression of pro-inflammatory cytokines in erosive inflammatory OA patients

Previous studies suggested that pro-inflammatory cytokines such as IL-1β, TNF-α and IL-6 play important roles in promoting the development of OA^10^. Their mRNA in PBMCs and protein produced by PBMCs were detected from erosive inflammatory OA and primary generalized OA patients under stimulation with or without human recombinant IL-37.

**Figure 3:**
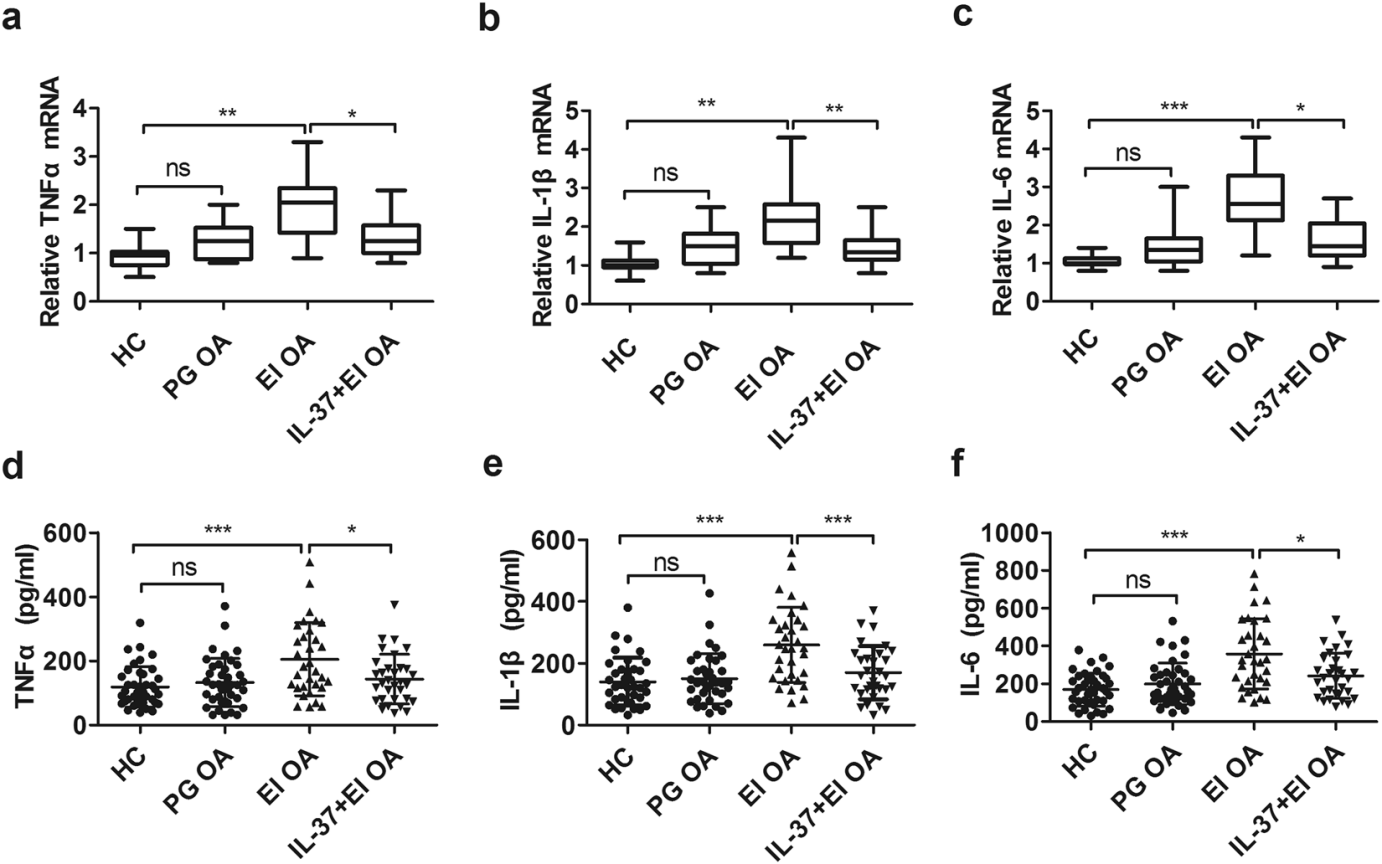
IL-37 suppresses the expression of pro-inflammatory cytokines in PBMCs from EIOA patients. The mRNAs expression of TNF-α **(a)**, IL-1β**(b)** and IL-6 **(c)** in PBMCs from EIOA patients (n = 32) and HCs (n = 40) were analyzed by RT-PCR, the supernatants produced by PBMCs were collected for measuring TNF-α**(d)**, IL-1β **(e)** and IL-6 **(f)** protein levels by ELISA.

Compared with HCs, the relative expression levels of IL-1β, TNF-α, and IL-6 transcripts were significantly up-regulated in erosive inflammatory OA subjects (Fig. 3a–c, p < 0.05), as were their protein levels (Fig. 3d–f, p < 0.05), but not in primary generalized OA patients. It is noteworthy that, the increased relative mRNA expression and protein levels of TNF-α (Fig. 3a,d, p < 0.05), IL-1β (Fig. 3b,e, p < 0.05), and IL-6 (Fig. 3c,f, p < 0.05) were dramatically inhibited in IL-37-treated PBMCs from EIOA patients.

### IL-37 significantly increased in synovial fluid and positive correlations with pro-inflammatory cytokines produced by synovial cells from EIOA patients

To further evaluate whether the expression of IL-37 was related to the inflammation of EIOA, we isolated synovial fluid and synovial cells from EIOA patients, the transcripts and synovial protein levels of IL-37 were examined in synovial cells. In addition, correlations between IL-37 and pro-inflammatory cytokines TNF-α, IL-1β and IL-6 were analyzed in synovial cells from 12 EIOA patients who undergone surgery.

**Figure 4:**
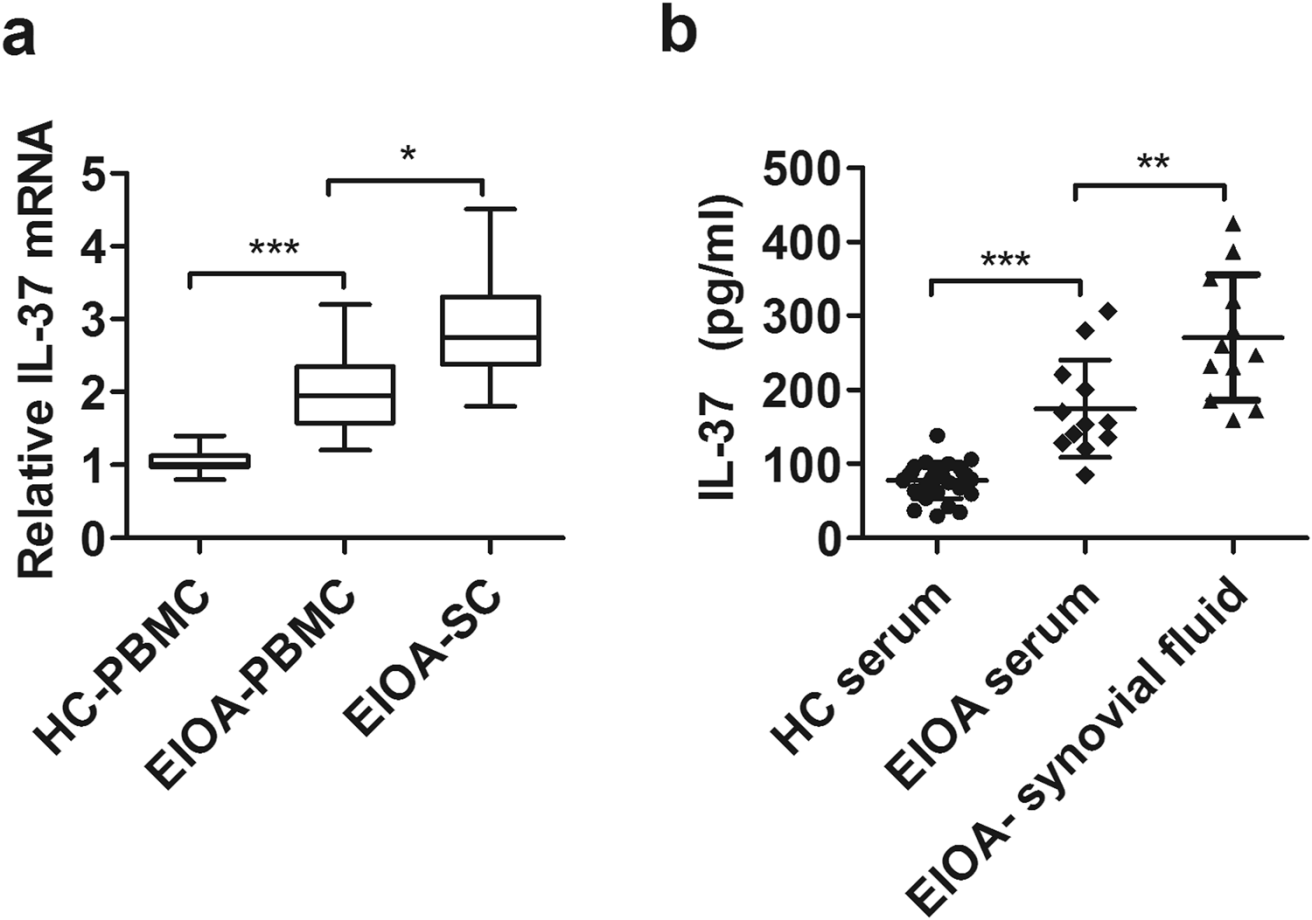
Comparison of IL-37 mRNAs and protein levels between PBMCs and Synovial cells (SCs) from EIOA patients. PBMCs and SCs were collected from EIOA patients (n = 12). the relative mRNA **(a)** and protein levels **(b)** were detected by RT-PCR and ELISA, respectively.

**Figure 5:**
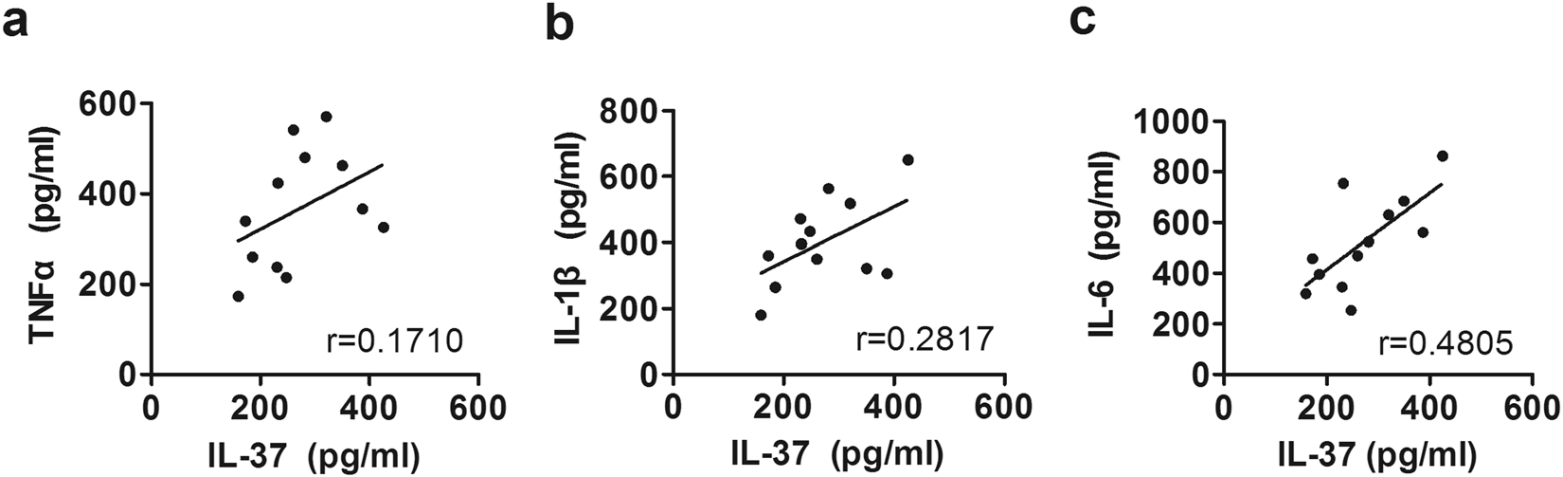
Correlation between IL-37 and pro-inflammatory cytokines in synovial cells from EIOA patients. Synovial IL-37 levels were positively correlated with TNF-α **(a)**, IL-1β **(b)**, and IL-6 **(c)** produced by SCs from EIOA patients (n = 12). Each symbol indicates an individual patient.

Compared with peripheral venous blood, IL-37 mRNA levels in synovial cells were elevated significantly in the same patients with erosive inflammatory OA (Fig. 4a, p < 0.05). Consistently, similar results were observed at protein levels (Fig. 4b, p < 0.05). Furthermore, a positive correlation was found between IL-37 and TNF-α (Fig. 5a r = 0.1710), IL-1β (Fig. 5b, r = 0.2817), IL-6 (Fig. 5c, r = 0.4805) produced by synovial cells.

### IL-37 inhibits the production of inflammatory cytokines in synovial cells from EIOA patients

Synovitis were reported to be involved in the development of OA^26, 27^, and the pro-inflammatory cytokines such as TNF-α, IL-1β and IL-6 produced by synovial cells played a significant role in the pathogenesis of OA^10, 28^. To investigate whether IL-37 suppresses the expression of these inflammatory cytokines in synovial cells, we isolated synovial tissues from EIOA patients who undergone surgery and cultured the synovial cells with or without recombinant IL-37 stimulation.

**Figure 6:**
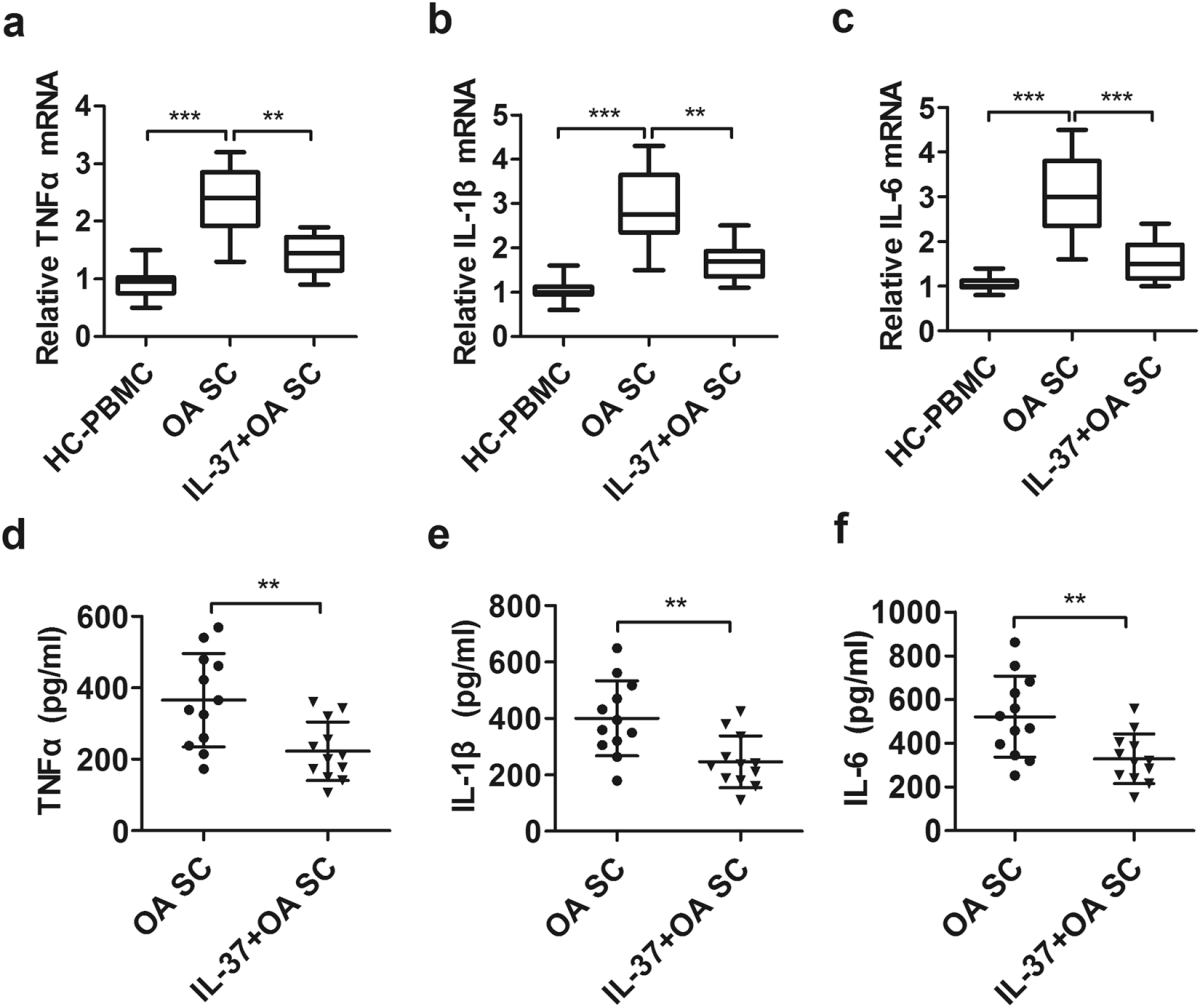
IL-37 inhibits the expression of inflammatory cytokines in synovial cells from EIOA patients. Synovial cells isolated from synovial tissues of EIOA patients (n = 12) were treated similarly to the PBMCs. The relative mRNA expression of TNF-α (**a**), IL-1β (**b**) and IL-6 (**c**) were analyzed by RT-PCR, the protein levels of these cytokines produced by SCs were analyzed by ELISA (**d**–**f**).

The results showed that the relative expression levels of TNF-α, IL-1β and IL-6 transcripts in synovial cells were significantly reduced by IL-37 treatment (Fig. 6a–c, p < 0.05). Consistently, similar results were observed at protein levels (Fig. 6d–f, p < 0.05), while the cell viability was unaltered upon IL-37 treatment during sample assessment (data now shown). These results further demonstrated that IL-37 could inhibit the expression of TNF-α, IL-1β and IL-6 in patients with EIOA. Together, these data indicate an anti-inflammatory effect of IL-37 in OA inflammation.

## Discussion

Our recent studies have shown that expression of IL-37 was increased in RA, SLE, GD and AS diseases and is correlated with activity of these diseases^22–25^. Although serum IL-37 level in RA patients has been demonstrated to be higher than the level of IL-37 in OA patients and healthy controls, the role played by IL-37 and its correlation with clinical characteristics of OA patients has remained largely unclear^29^. Here, we provide a detailed analysis of IL-37expression in EIOA and PGOA patients, and reveal the inhibiting effect of IL-37 in synovial inflammation of osteoarthritis.

In this study, increased transcription and protein levels of IL-37 in blood from EIOA patients were demonstrated when compare to that of PGOA patients and HCs (Fig. 1).In addition, significantly positive correlations were observed between IL-37 level in serum and clinical VAS score, CRP, ESR (Table S2). So our results indicated that IL-37 is increased in EIOA and closely related to OA inflammation.

OA is considered as a wear and tear disease but not inflammatory arthropathy in the past, because of the presence of a small number of neutrophils in the synovial fluid^30^. However, current studies have indicated that the disease symptoms, such as joint pain, swelling and stiffness, are attributable to inflammation in OA^5^. The crucial cytokines IL-1β and TNF-α promoted the production of pro-inflammatory cytokines, such as IL-6, which in turn synergized with IL-1β and TNF-α to boost the inflammatory response in OA pathologic conditions^10^. These cytokines are partly responsible for the occurrence of swelling and pain in human EIOA^9, 31–33^. Therefore, TNF-α, IL-1β, and IL-6 were detected in PBMCs from OA patients and HCs, the relative mRNA expression and protein levels of these inflammatory cytokines are significantly higher in EIOA patients than in PGOA patients and HCs (Fig. 3). Consistent with previous findings^10, 11^, these results further suggested that markedly increased TNF-α, IL-1β and IL-6 were closely related to OA pathogenesis.

To further explore the relationship between IL-37 and EIOA disease, we isolated synovial fluid and synovial cells from EIOA patients with knee surgery. It is found that significant increased IL-37 expression in synovium compare to blood from EIOA patients (Fig. 4). In addition, positive correlations were observed among IL-37 with TNF-α, IL-1β and IL-6 in synovial cells from EIOA patients (Fig. 5 and Table S3). These findings indicated that the IL-37 levels may be closely related to the levels of pro-inflammatory cytokines in EIOA. This phenomenon can be explained by published study which showed that IL-37 can be induced by pro-inflammatory cytokines *in vitro* ^21^. Our assumption is that EIOA patients have higher levels of pro-inflammatory cytokines such as TNF-α, IL-1β, and IL-6, which act as positive feedback loops for increasing the production of IL-37.

It has been widely accepted that IL-37 acts as a fundamental inhibitor of innate immunity and plays an important role in the regulation of inflammatory cytokines in various inflammatory diseases^17–25^. IL-37 was recognized to suppress immune responses by shifting the cytokine equilibrium away from excessive inflammation^21^. Our published studies also showed that IL-37 act as a negative regulator, suppressing inflammatory responses in a number of autoimmune diseases^22–25^. In this study, ours results indicated that the mRNA and protein levels of TNF-α, IL-1β, and IL-6 in PBMCs from EIOA patients were markedly increased, which can be significantly restrained by IL-37 recombinant protein *in vitro* (Fig. 3). Consistent with our previously published data, these results indicate that IL-37 acts as an immune suppressor of pro-inflammatory cytokines in PBMCs from EIOA patients.

Synovitis occurred frequently in osteoarthritis^33^, along with the increasing pro-inflammatory cytokines, such as TNF-α, IL-6 and IL-1β in synovial tissue, played a pivotal role in the development of OA inflammation^34–36^. Therefore, it is necessary to investigate whether the protective functions of IL-37 extend to synovial cells. Therefore, we pretreated synovial cells with recombinant IL-37 protein, which was found to effectively inhibit the production of TNF-α, IL-1β and IL-6 in synovial cells from EIOA patients (Fig. 6). These results indicate that IL-37 can directly inhibit the inflammatory response in synovial cells, further confirming the anti-inflammatory effect of IL-37 in patients with EIOA.

In this study, we provide a new insight for unraveling the interesting balance between IL-37 and pro-inflammatory cytokines in EIOA. Since the anti- inflammatory activity of IL-37 required the active involvement of IL-1R8 and IL-18Ra, which assembling with IL-37 formed tripartite complex to activate a multifaceted intracellular anti-inflammatory program^37^, further studies focusing on the molecular mechanism elucidate the IL-37-mediated immunosuppression in OA.

## Conclusion

In summary, our study showed that the endogenous IL-37 increased in PBMCs of EIOA patients is not only associated with VAS score but also positively correlated with CRP, ESR. The pro-inflammatory cytokines in OA, such as TNF-α, IL-1β and IL-6, which participate in the pathogenesis of EIOA, are positively correlated with IL-37. Importantly, our experiments indicated that these pro-inflammatory cytokines in PBMCs and synovial cells from EIOA patients could be suppressed by IL-37 *in vitro*. These data indicate that IL-37 plays an important role in protecting EIOA from excessive inflammatory response. Therefore, IL-37 may provide a novel treatment target for OA inflammation. However, further studies are needed to elucidate the regulatory mechanisms of IL-37 in the pathogenesis of EIOA.

## Methods

### Ethics Statement

This study was approved by the Ethics Committee of Shenzhen People’s Hospital, China. All methods were carried out in accordance with the approved guidelines. Written, informed consent was obtained from gout patients and healthy controls who participated in this study. All participants were informed of their right to discontinue participation at any time.

### Patients and controls

Seventy-two OA patients with informed consent were recruited from the Rheumatology and Immunology Department, Shenzhen People’s Hospital. Each patient diagnosed as OA in line with the classification developed by the Osteoarthritis Criteria Subcommittee of the American Rheumatism Association^38^. According to X- ray or MRI diagnosis and clinical parameters, Patients with OA can be distinguished erosive inflammatory OA from primary generalized OA^7^. Patients with other rheumatic diseases, infections or malignant tumors were excluded from the study. Clinical data from each OA patient were recorded, visual analogue scale (VAS) was used to measure pain intensity, with lower scores(0~10) indicating lower levels of clinical symptoms or pain^39^. Other clinical data containing C-reactive protein (CRP), erythrocyte sedimentation rate (ESR), albumin (ALB), urea nitrogen (BUN) Cr (Creatinine), anti-CENPB antibody and anti-SSA60 antibody were performed. Forty age and sex matched healthy volunteers were recruited from the same hospital as controls (HCs). Demographic and clinical information were listed in Table 1.

**Table 1:** Demographic and clinical characteristics of OA and healthy controls.

Characteristics	OA patients (n = 72)	Healthy controls (n = 40)
Age (years)	63.89 ± 14.34	62.32 ± 14.15
Sex (male/female)	12/60	8/32
Disease duration (years)	4.56 ± 3.9	—
Anti-CenpB n (%)	6(8.3)	—
Anti-SSA60 n (%)	5(7)	—
ALB (g/L)	39.20 ± 5.16	—
ESR (mm/h)	29.53 ± 24.14	< 20
CRP (mg/L)	7.49 + ± 7.12	< 5
BUN (mmol/L)	5.58 ± 2.31	—
Cr (μmol/L)	94.96 ± 46.60	—
VAS	5.58 ± 2.31	—

### Sample collection and cell isolation

Blood samples were collected from venous blood in the morning from Shenzhen People’s Hospital. PBMCs were isolated from OA patients and healthy controls within three hours by a Ficoll-Paque plus (TBD science, China) density gradient centrifugation under sterile conditions. Synovial fluid and synovial tissues from 12 knee osteoarthritis patients with erosive inflammation were collected during surgeries, synovial cells (SCs) were immediately isolated from synovial tissues according to the method described in ref. 40. These isolated cells were used for RNA extraction or cell culture for treatment.

### Recombinant human IL-37 protein

Both expression and purification of human recombinant IL-37 protein, were described in our previous study^22^. The recombinant protein concentrations were detected by the Bradford method and preserved at −80 °C.

### Cell culture condition

PBMCs and SCs were cultured with RPMI 1640 (HyClone, Thermo, USA) medium enriched with 10% fetal calf serum (HyClone, Thermo, USA),100 IU/ml penicillin, 100 μg/ml streptomycin with a density of 1 × 10^6^cells/ml in 12-well plates. These cells were treated under identical conditions, with or without human recombinant IL-37 100 ng/ml for 12 h, and stimulated with LPS (1 μg/ml) for 4 h. Subsequently, total RNA extracted from these cells and transcriptions levels of pro-inflammatory cytokines were analyzed by RT-PCR. For detecting the protein levels of these cytokines in culture supernatants, PBMCs and SCs were stimulated with or without human recombinant IL-37 100 ng/ml for 24 h and then incubated further with LPS (1 μg/ml) for 4 h. Supernatants were then collected and frozen at −80 °C for later analysis by ELISA.

### RNA extraction and RT-PCR

Total RNA extraction and cDNA synthesis were performed as described previously^24^. The primer sequences used for RT-PCR in this study are shown in Table S1. The relative expression of target genes IL-37, TNF-α, IL-1β and IL-6 was normalized to control housekeeping genes GAPDH and were reported using the 2^−ΔΔct^ method.

### ELISA

Serum IL-37, TNF-α, IL-1β and IL-6 were quantified by platinum ELISA kits (San Diego, CA, USA). Detection of the levels of the cytokines TNF-α, IL-1β, and IL-6 in cell culture supernatant was performed using the eBioscience kit (San Diego, USA) following the manufacturer’s instructions.

### Statistical analysis

The results are presented as the mean ± SD. Spearman correlations test were used to assess the association between serum IL-37 levels and OA clinical, as well as the correlations of IL-37 in synovial fluid with pro-inflammatory cytokines in SCs. Comparisons between groups were determined by using nonparametric Mann-Whitney U-test. Ns indicate no significant difference. p < 0.05, p < 0.01 and p < 0.001 in figures were considered statistically significant and represent as *, ** and ***, respectively. Statistical analysis was performed with Graph Pad Prism 5.0 software (San Diego CA, USA).

## Electronic supplementary material

supplementary information
