# Supplementary material for: IL-37 is associated with osteoarthritis disease activity and suppresses proinflammatory cytokines production in synovial cells
Source: Sci Rep. 2017 Sep 14;7:11601. doi: 10.1038/s41598-017-11397-5 (PMC5599693; doi:10.1038/s41598-017-11397-5)
Supplement: Supplementary file 1 — supplementary information [file 41598_2017_11397_MOESM1_ESM.pdf]

IL-37 is associated with osteoarthritis disease activity and suppresses proinflammatory cytokines production in synovial cells

Liping Ding [1†], Xiaoping Hong[1†], Baodong Sun[1], Qin Huang[1], Xiaoqi Wang[1], Xiaokai Liu[2], Lingyun Li[2], Zhong Huang[2*], Dongzhou Liu[1*]

[†] Equal contributors

[1] Department of Rheumatology and Immunology, Shenzhen People's Hospital, the Second Clinical Medical College of Jinan University, Shenzhen, China.

[2] Department of Immunology and Microbiology, Biological Therapy Institute, Shenzhen University School of Medicine, Shenzhen, China.

*** Address and correspondence to:**

Dongzhou Liu, chief physician, professor, MD

Department of Rheumatology and Immunology

Shenzhen People's Hospital, Second Clinical Medical College of Jinan University

No. 1017 Dongmen North Road, Shenzhen, China

Phone: +86-13802257360

Fax: +86-0755-22943519

*****Co-correspondence: zhuang809@126.com; liu_dz2001@sina.com

Table S1. List of the sequence of human gene primers

Gene name	Forward (5′to 3′)	Reverse (5′to 3′)
IL-37	AGTGCTGCTTAGAAGACCCGG	AGAGTCCAGGACCAGTACTTTGTGA
TNF-α	CTTCTCGAACCCCGAGTGAC	TGAGGTACAGGCCCTCTGATG
IL-1β	GCGGCCAGGATATAACTGACTTC	TCCACATTCAGCACAGGACTCTC
IL-6	AGCCACTCACCTCTTCAGAAC	ACATGTCTCCTTTCTCAGGGC
GAPDH	TGCACCACCAACTGCTTAGC	GGCATGGACTGTGGTCATGAG

Table S2. Correlation between serum IL-37 levels and clinical laboratory values

Parameter	Correlation coefficient (r)	*p*-value
VAS Score	0.2954	<0.0001
CRP (mg/L)	0.1570	0.0006
ESR (mm/h)	0.1839	0.0002

Table S3. Correlation between synovial fluid IL-37 and inflammatory cytokines produced by synovial cells from EIOA patients

Parameter	Correlation coefficient (r)	*p*-value
TNF-α	0.1710	0.1815
IL-1β	0.2817	0.0758
IL-6	0.4805	0.0124
